# Cortical Thickness Changes Correlate with Cognition Changes after Cognitive Training: Evidence from a Chinese Community Study

**DOI:** 10.3389/fnagi.2016.00118

**Published:** 2016-05-24

**Authors:** Lijuan Jiang, Xinyi Cao, Ting Li, Yingying Tang, Wei Li, Jijun Wang, Raymond C. Chan, Chunbo Li

**Affiliations:** ^1^Shanghai Key Laboratory of Psychotic Disorders, Shanghai Mental Health Center, Shanghai Jiao Tong University School of MedicineShanghai, China; ^2^Bio-X Institutes, Key Laboratory for the Genetics of Developmental and Neuropsychiatric Disorders, Ministry of Education, Shanghai Jiao Tong UniversityShanghai, China; ^3^Brain Science and Technology Research Center, Shanghai Jiao Tong UniversityShanghai, China; ^4^Shanghai Chang Ning Mental Health CenterShanghai, China; ^5^Neuropsychology and Applied Cognitive Neuroscience Laboratory, Key Laboratory of Mental Health, Institute of Psychology, Chinese Academy of SciencesBeijing, China

**Keywords:** aging, cognitive function, cortical thickness, multi-domain, single-domain

## Abstract

The aim of this study was to investigate whether changes in cortical thickness correlated with cognitive function changes in healthy older adults after receiving cognitive training interventions. Moreover, it also aimed to examine the differential impacts of a multi-domain and a single-domain cognitive training interventions. Longitudinal magnetic resonance imaging (MRI) scanning was performed on participants 65–75 years of age using the Siemens 3.0 T Trio Tim with the Magnetization Prepared Rapid Gradient Echo (MPRAGE) sequence. The cortical thickness was determined using FreeSurfer Software. Cognitive functioning was evaluated using the Repeatable Battery for the Assessment of Neuropsychological Status (RBANS). There were significant group × time interaction effects on the left supramarginal, the left frontal pole cortical regions; and a marginal significant group × time interaction effects on visuospatial/constructional and delayed memory scores. In a multi-domain cognitive training group, a number of cortical region changes were significantly positively correlated with changes in attention, delayed memory, and the total score, but significantly negatively correlated with changes in immediate memory and language scores. In the single-domain cognitive training group, some cortical region changes were significantly positively associated with changes in immediate memory, delayed memory, and the total score, while they were significantly negatively associated with changes in visuospatial/constructional, language, and attention scores. Overall, multi-domain cognitive training offered more advantages in visuospatial/constructional, attention, and delayed memory abilities, while single-domain cognitive training benefited immediate memory ability more effectively. These findings suggest that healthy older adults benefit more from the multi-domain cognitive training than single-domain cognitive training. Cognitive training has impacted on cortical thickness changes in healthy elderly.

## Introduction

As the average human lifespan increases and the world’s aging population grows, issues surrounding the health and care of the aging are gaining increasing attention (Hu et al., 2014). Cognitive decline associated with aging, especially in episodic memory, attention, and executive functions, has been reported in both longitudinal (Meijer et al., 2009) and cross-sectional studies (Coubard et al., 2011; Kobayashi et al., 2015). As a means of prevention and treatment, cognitive training is designed to restore, increase, or optimize capacities in persons with cognitive decline or normal aging (Thompson and Foth, 2005; Belleville and Bherer, 2012; Rebok et al., 2014). Single-domain cognitive training focuses either on memory (Ball et al., 2002; Sisco et al., 2013), reasoning (Payne et al., 2012), strategy training (Kirchhoff et al., 2012) or processing speed (Cody et al., 2015). While multi-domain cognitive training combines several cognitive functions and demands their interplay (Corbett et al., 2015; Rahe et al., 2015). Beneficial changes at the behavioral level in cognitive function, as well as the structural level in the aging brain, have been proven possible as the result of cognitive training (Lustig et al., 2009; Reijnders et al., 2013; Rahe et al., 2015).

A number of randomized controlled trials (RCTs) have shown that cognitive training can improve cognitive performance in healthy older adults (Engvig et al., 2010; Mozolic et al., 2011; Kwok et al., 2013; Sisco et al., 2013; Corbett et al., 2015). For instance, Kwok et al. (2013) reported that the Active Mind cognitive-training program was effective in improving the cognitive function and quality of life for community-dwelling Chinese older adults in Hong Kong. Sisco et al. (2013) demonstrated that multifactorial memory training can improve the verbatim recall of prose in healthy older adults aged 65–91. Online cognitive training package (including memory, reasoning, attention training) in older adults has shown benefited to reasoning and verbal learning (Corbett et al., 2015).

Many studies have relied on structural brain imaging, such as whole-brain volume, regional gray matter volumes, and cortical thickness, to assess the effect of cognitive training in aging patients. The cortex, which is a tightly folded sheet of neurons, ranges in thickness between 1.5 and 4.5 mm (Parent and Carpenter, 1995). Measurements of cortical thickness can reflect the size, density, and arrangement of cells, which may be more closely linked with cognition ability than volumetric or intensity-based gray matter (GM) concentration measures, although these have also been shown to relate to other local measures of GM (Narr et al., 2007). Therefore, measurements of cortical thickness could provide important information about the regional integrity of the cerebral cortex and supply new insights into cognitive training (Liu et al., 2014).

The relationship between brain volumes and cognitive functions has been long-standing research interest. For example, some researchers have found positive correlations between GM volume and executive functions (Newman et al., 2007; Ruscheweyh et al., 2013) in cognitively healthy adults, and memory impairment is often associated with the atrophy of limbic structures (including the hippocampus and parahippocampal gyrus; Jack et al., 1992). However, fewer have shown the cortical thickness is correlated with cognitive functions (Ehrlich et al., 2012; Velayudhan et al., 2013; Kim et al., 2015b). For instance, Velayudhan et al. (2013) found that reduced thickness of the entorhinal cortex was related to reduced scores on perceptual and executive function tests, including perceptual speed and aspects of working memory in Alzheimer’s disease. In another study, right parahippocampal gyrus atrophy showed a significant correlation with the executive and visuospatial functions impairment in Parkinson’s disease (Kim et al., 2015b).

Taken together, we hypothesized that cortical thickness changes would significantly associated with changes in cognition, thereby revealing more brain mechanism of the cognitive training effect on healthy older adults. However, to our knowledge, no randomized, controlled study comparing multi-domain and single-domain cognitive training interventions in healthy older adults has been performed prior to our study. We also evaluated the effects of multi-domain and single-domain cognitive training interventions in healthy older adults.

## Materials and Methods

### Subjects

Participants were community-dwelling elders living in a neighborhood located in the Putuo District of Shanghai from March 2008 to April 2008. Inclusion criteria mandated that participants: (a) were 65–75 years of age; (b) had no severe physical or mental illnesses (such as brain tumor, cerebral infarction, cerebral hemorrhage, malnutrition, major depressive disorder and schizophrenia); (c) were able to live independently; (d) had no disability, and no difficulties in hearing, vision or communication; (e) had at least 1 year of formal education; and (f) had an education-adjusted normal score on the Chinese version of the Mini-Mental State Examination (MMSE; Li et al., 2006; >14 for those who had not finished primary school; >19 for those who completed primary school; or >24 for those who had graduated from middle school). Any participants exhibiting obvious cognitive decline, or who had received a diagnosis of Alzheimer’s disease or other major medical conditions, were excluded.

All participants underwent magnetic resonance imaging (MRI) scanning and cognitive assessments at baseline (Time 1) and a 12-month follow-up after the intervention (Time 2). Participants who were absent for one MRI scanning or one cognitive assessment were excluded from data analysis. All participants in this study provided a written informed consent form (LL(H)-09-04). This study was approved by the Human Research Ethics Committee of Tongji Hospital in Shanghai, China.

### Cognitive Training Program

The cognitive training intervention was delivered by a trained expert in small groups (*n* ≤ 15). Each participant from the groups attended a total of 24 sessions, with each session being 60 min long, 2 days a week, for 12 weeks. The multi-domain cognitive training consisted of activities geared towards memory, reasoning, problem solving strategies, visuospatial map reading skills development, handcraft making, and health and physical exercise; the single-domain cognitive training specifically targeted reasoning training, including the Tower of Hanoi, numerical reasoning, Raven Progressive Matrices, and verbal reasoning. Booster training was provided to each group, which included one additional 60-min training session every month, from the 6-month follow-up to the 9-month follow-up after the intervention. A detailed description of the program was provided in the previous publication (Cheng et al., 2012).

### Cognitive Assessment

The Repeatable Battery for the Assessment of Neuropsychological Status (RBANS, Form A; Randolph et al., 1998) was administered to participants in order to assess cognitive functioning. It consists of 12 tasks that measure five domains of cognitive functioning: (a) immediate memory was measured using a list learning task (40 points) and a story memory task (24 points); (b) visuospatial/constructional ability was measured using a figure copy task (20 points) and a line orientation task (20 points); (c) language was measured using a picture naming task (10 points) and a semantic fluency task (40 points); (d) attention was measured using a digit span task (16 points) and a coding task (89 points); and (e) delayed memory was measured using a list recall task (10 points), a list recognition task (20 points), a story recall task (12 points), and a figure recall task (20 points). The RBANS includes six scores: total scale score and five index scores. Administration and scoring of the RBANS in this study were conducted by trained personnel according to standardized instructions (Randolph et al., 1998). RBANS (Form A) had good validity and reliability in a Chinese community-living elderly sample (Lim et al., 2010; Cheng et al., 2011).

### Image Acquisition

Structural MRI scans were performed using a Siemens Tim Trio 3T scanner (Siemens Medical Solutions, Erlangen, Germany) with a 12 channel head coil. The pulse sequence used for morphometric analyses was three-dimensional T1-weighted Magnetization Prepared Rapid Gradient Echo (MPRAGE), with the following parameters: repetition time (TR) = 1900 ms, echo time (TE) = 3.43 ms, flip angle = 9°, image matrix = 256 × 256 mm, field of view (FOV) = 24 cm. Each scan took 5 min. Each volume consisted of 160 sagittal slices with a 1.0 mm slice thickness, voxel size = 0.9 × 0.9 × 1.0 mm^3^. Images were reconstructed and visually checked for major artifacts (e.g., motion, ringing, wrap around and neurological abnormalities) before further processing.

### Cortical Thickness Analysis

For the image analysis, we used the FreeSurfer software package 5.3.0^1^ for Mac OS. To estimate cortical thickness, images were automatically processed with the longitudinal stream in FreeSurfer (Reuter et al., 2012). All scans from two time points (Time 1 and Time 2) were first preprocessed independently with the cross-sectional stream. Then, each subject’s base template was created from the scans of his or her two time points, which operates as an initial estimate for the segmentation and surface reconstruction. The two measurement time points were then registered to the base template in order to ensure non-biased analysis with regard to the two time points (Wenger et al., 2012). All reconstructed data were visually checked for segmentation accuracy at each time point. This work flow has been shown to significantly increase reliability and statistical power (Han et al., 2006). Based on gyral and sulcal anatomy, 34 distinct cortical regions were then segmented for each hemisphere, using the Desikan–Killiany Atlas (Desikan et al., 2006). Finally, the cortical thickness was measured as the shortest distance (in mm) between the white matter surface and the pial surface (Fischl and Dale, 2000).

### Statistical Analysis

All statistical analyses were performed using the Statistical Package for the Social Sciences (SPSS; SPSSInc., Chicago, IL, USA) version 17.0 for Windows. The statistical significance threshold was set at *p* < 0.05. A repeated-measures analysis of variance (ANOVA) was performed to evaluate the training effect on RBANS scores and cortical thickness. Correlation between the RBANS change scores and cortical thickness changes in the participants using partial correlation after correcting for age, gender, years of education, and estimated total intracranial volume (eTIV). We computed RBANS change scores (Time 2 minus Time 1), as well as cortical thickness changes (Time 2 minus Time 1). We analyzed the multi-domain and single-domain cognitive training groups separately.

## Results

### Characteristics of Study Participants

The demographic and cognitive outcomes of healthy older adults at baseline and 12-month follow-up are shown in Table 1. At baseline, 24 subjects from the multi-domain training group and 24 subjects from the single-domain training group underwent cognitive assessment and the MRI scanning. Eighteen of the multi-domain training group and 18 of the single-domain training group finished the cognitive assessment and MRI scanning at 12-month follow-up after the intervention. A total of 12 participants withdrew, two due to left-handedness, one due to death, two due to intestinal cancer, one due to her husband’s death, two due to operation and four due to rejecting the scanning. There were no significant differences between groups in terms of age, gender, or any cognitive outcome measurements, except for the years of education (*t* = 2.63, *p* = 0.013) at the 12-month follow-up. Due to some participants missing one MRI scanning or one cognitive assessment at the 12-month follow-up, these results were excluded from data analysis, thus leading to the single-domain training group demonstrating a lower figure for years of education than the multi-domain training group at the 12-month follow-up.

**Table 1:** **Characteristics of demographic information and cognitive outcomes**.

		Multi-domain training group	Single-domain training group	*t*/χ^2^	*p*
Age	Baseline	70.54 ± 3.23	70.00 ± 3.90	0.52	0.603
	12-month follow-up	71.33 ± 3.31	71.17 ± 3.87	0.14	0.890
Years of education	Baseline	10.83 ± 3.60	9.08 ± 4.06	1.58	0.121
	12-month follow-up	11.67 ± 3.20	8.39 ± 4.22	2.63	0.013*
Gender (male:female)	Baseline	17:7	12:12	2.18	0.140
	12-month follow-up	13:5	8:10	2.86	0.091
RBANS total score	Baseline	90.13 ± 16.01	92.13 ± 14.12	−0.46	0.648
	12-month follow-up	106.94 ± 12.90	102.50 ± 16.30	0.91	0.371
Immediate memory	Baseline	84.17 ± 15.38	85.88 ± 15.47	−0.38	0.703
	12-month follow-up	103.17 ± 23.35	101.61 ± 18.67	0.22	0.827
Visuospatial/Constructional	Baseline	103.88 ± 15.59	101.79 ± 11.28	0.53	0.598
	12-month follow-up	103.94 ± 11.56	103.50 ± 17.24	0.09	0.928
Language	Baseline	92.46 ± 13.47	93.83 ± 11.01	−0.39	0.700
	12-month follow-up	101.00 ± 8.39	99.11 ± 8.47	0.67	0.506
Attention	Baseline	86.08 ± 20.02	90.63 ± 15.81	−0.87	0.388
	12-month follow-up	94.94 ± 15.17	94.94 ± 18.57	0.00	1.000
Delayed memory	Baseline	96.54 ± 16.87	99.21 ± 20.11	−0.50	0.621
	12-month follow-up	118.50 ± 12.41	109.56 ± 19.79	1.63	0.113

*p < 0.05 significant two-sided testing.

### The Effect of Training on Cortical Thickness and Cognition

**Figure 1:**
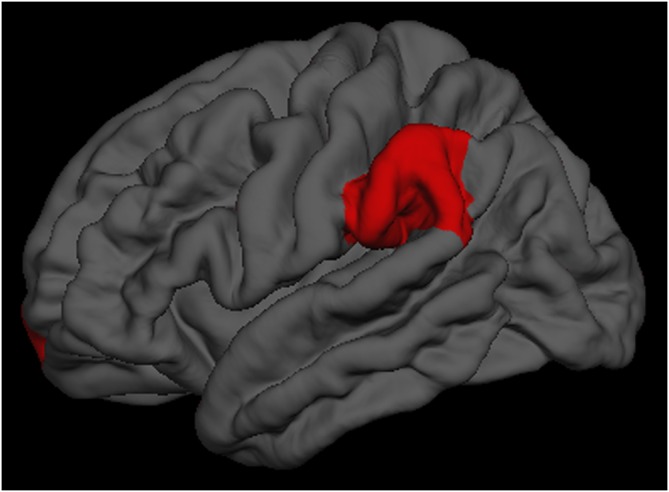
**The left supramarginal and left frontal pole regions had significantly group × time interaction effects**.

To investigate the impact of the cognitive training on the cortical thickness, the repeated-measures ANOVA showed a significant group × time interaction effects on the left supramarginal region (*p* = 0.025) and the left frontal pole region (*p* = 0.040; Figure 1). Tests of simple main effects revealed that the left supramarginal region (*p* = 0.020) cortical thickness was significantly reduced comparing to baseline in the single-domain training group, but not in the multi-domain training group. When the analyses were to investigate the impact of training on cognitive scores, a repeated-measures ANOVA revealed a marginal significant group × time interaction effects on visuospatial/constructional scores (*p* = 0.081) and delayed memory scores (*p* = 0.060). However, there were no group × time interaction effects on the other cortical regions and cognitive scores.

### Associations of Cortical Thickness and Cognition

As shown in Tables 2, 3, the cortical thickness changes associated with specific cognitive functions change scores are somewhat different in the multi-domain and single-domain training groups. Table 2 presents the correlations between cognitive function change scores and cortical thickness changes in the multi-domain training group, after controlling for age, gender, education, and eTIV. It shows that the immediate memory change scores were significantly negatively associated with the changes in the left parahippocampal and right transverse temporal regions. In this group, visuospatial/constructional change scores were not significantly associated with any of the cortical thickness changes. However, significant negative correlations between language change scores and cortical thickness changes were observed in the change of the left parahippocampal region, and significant positive correlations between the attention change scores and cortical thickness changes were indicated in the change of the right frontal pole region. Furthermore, significant positive correlations between delayed memory change scores and cortical thickness changes were presented in the changes of the left inferior parietal, lateral occipital, lingual, superior parietal, and supramarginal, as well as the right cuneus, inferior parietal, lateral occipital, lateral orbitofrontal, pars orbitalis, posterior cingulate, rostral anterior cingulate, and insula regions. Finally, significant positive correlations between total change scores and cortical thickness changes were observed in changes to the left superior parietal and the right lateral occipital, temporal pole regions.

**Table 2:** **Partial correlations between repeatable battery for the assessment of neuropsychological status (RBANS) change scores and cortical thickness changes in the multi-domain training group, by controlling for age, gender, education, and estimated total intracranial volume (eTIV)**.

	Immediate memory		Language		Attention		Delayed memory		Total score	
Cortical regions	*r*	*p*	*r*	*p*	*r*	*p*	*r*	*p*	*r*	*p*
**Left hemisphere**
Inferior parietal							0.552	0.040*
Lateral occipital							0.546	0.044*
Lingual							0.570	0.033*
Parahippocampal	−0.582	0.029*	−0.616	0.019*
Superior parietal							0.776	0.001*	0.608	0.021*
Supramarginal							0.578	0.030*
**Right hemisphere**				
Cuneus							0.609	0.021*
Inferior parietal							0.565	0.035*
Lateral occipital							0.732	0.003*	0.554	0.040*
Lateral orbitofrontal							0.609	0.021*
Pars orbitalis							0.650	0.012*
Posterior cingulate							0.652	0.012*
Rostral anterior cingulate							0.561	0.037*
Frontal pole					0.636	0.015*		
Temporal pole									0.605	0.022*
Transverse temporal	−0.606	0.022*		
Insula							0.550	0.042*

RBANS, Repeatable Battery for the Assessment of Neuropsychological Status; eTIV, estimated total intracranial volume; *p < 0.05 significant two-sided testing.

**Table 3:** **Partial correlations between RBANS change scores and cortical thickness changes in the single-domain training group, by controlling for age, gender, education, and eTIV**.

	Immediate memory		Visuospatial/Constructional		Language		Attention		Delayed memory		Total score	
Cortical regions	*r*	*p*	*r*	*p*	*r*	*p*	*r*	*p*	*r*	*p*	*r*	*p*
**Left hemisphere:**
Entorhinal									0.608	0.021*
Paracentral			−0.697	0.006*
Pars orbitalis									0.710	0.004*
Posterior cingulate									0.646	0.013*
Precuneus									0.645	0.013*
Rostral anterior cingulate									0.543	0.045*
Supramarginal	0.557	0.039*							0.722	0.004*
**Right hemisphere:**						
Entorhinal	0.595	0.025*				
Lateral occipital							−0.601	0.023*		
Lingual							−0.605	0.022*		
Paracentral			−0.542	0.045*		
Pericalcarine							−0.584	0.028*		
Posterior cingulate									0.556	0.039*
Precentral							−0.598	0.024*		
Precuneus									0.589	0.027*
Rostral anterior cingulate					−0.647	0.012*
Superior parietal									0.662	0.010*
Frontal pole											0.585	0.028*

RBANS, Repeatable Battery for the Assessment of Neuropsychological Status; eTIV, estimated total intracranial volume; *p < 0.05 significant two-sided testing.

Table 3 presents the correlations between cognitive function change scores and cortical thickness changes in the single-domain training group, after controlling for age, gender, education, and eTIV. These results revealed that immediate memory change scores were significantly positively associated with the changes of the left supramarginal and the right entorhinal regions. Visuospatial/constructional change scores were significantly negatively associated with the changes of the left paracentral and the right paracentral regions and significant negative correlations between language change scores and cortical thickness changes were observed in the change of the right rostral anterior cingulate region. Attention change scores were significantly negatively correlated with the changes of the right lateral occipital, lingual, pericalcarine, and precentral regions. Significant positive correlations between delayed memory change scores and cortical thickness changes were presented in the changes of the left entorhinal, pars orbitalis, posterior cingulate, precuneus, rostral anterior cingulate, and supramarginal, as well as the right posterior cingulate, precuneus, and superior parietal regions. Lastly, significant positive correlations between total change scores and cortical thickness changes were observed in the change of the right frontal pole region.

## Discussion

The main finding of this study was that the cortical thickness changes were significantly associated with cognitive function changes in healthy elderly after receiving cognitive training interventions, and healthy older adults benefited more from the multi-domain cognitive training than single-domain cognitive training. Single-domain training targets highly specific cognitive abilities, it may allow researchers to evaluate direct training-related effects (Cheng et al., 2012). However, multi-domain training may have more practical advantages for older adults because that will increase engagement and transfer (Binder et al., 2015; Kim et al., 2015a).

There were significantly group × time interaction effects on the left supramarginal and the left frontal pole cortical regions, which two regions were parts of attention network (Fan et al., 2005; Grant et al., 2013). These findings implicated that the multi-domain cognitive training would offer more advantages in attention in the healthy older adults. Due to the relative small sample size, the results from the repeated-measures ANOVA may lead to an underestimation of interaction effects, the visuospatial/constructional scores and delayed memory scores showed a marginal significant group × time interaction effects. As such, cognitive training could delay age-associated cortical change and cognitive decline, it was an efficacious behavioral strategy for improving or maintaining cognitive health in the healthy elderly (Kim et al., 2015a; Smith-Ray et al., 2015).

In our study, both the multi-domain and single-domain training groups demonstrated delayed memory change scores that were significantly positively correlated with many distinct cortical region changes. These distinct cortical regions exhibited normal functions that were directly involved in improving delayed memory. This result showed that cognitive training can improve delayed memory in older persons living in a community.

As indicated previously, changes in brain structure, as well as cognitive functions, are commonly seen in the aging population. The human brain is a complex network of functionally and structurally interconnected regions. For example, the attention network contains the integrated regions of the frontal lobe, superior parietal lobe, fusiform gyrus, anterior cingulated, thalamus, and left precentral gyrus (Fan et al., 2005). Similar to previous studies (Fan et al., 2005), we observed significantly positive correlations between changes in attention scores and right frontal poles in the multi-domain training group. However, in the single-domain training group, attention change scores were significantly negatively correlated with change in the right lateral occipital, lingual, pericalcarine, and precentral. Cognitive function in aging does not have a single etiology, as there is not one exclusive brain region or system that is affected. According to a compensation view (Grady, 2012; Walhovd et al., 2014; Morcom and Johnson, 2015), it may be that the right lateral occipital, lingual, pericalcarine, and precentral regions are indirectly involved in improving attention, and require other brain regions to be involved in the process. Our analysis suggests that multi-domain training was more beneficial to attention improvement than single-domain training in healthy older adult participants. In accordance with a compensation view, we also concluded that multi-domain cognitive training was more effective in improving visuospatial/constructional skills, and single-domain cognitive training was more effective in improving immediate memory.

There are several limitations to this study that should be kept in mind when interpreting our results. The first major limitation involves the relatively conservative number of subjects, as we only included a small proportion of all identified community members who met the criteria for the study. Larger sample size is needed to evaluate the effect of cognitive training on the aging brain. Second, the multi-domain training group had a slightly higher education level than the single-domain training group at the 12-month follow-up (as explained in “Results” Section). However, years of education was used as covariate in our analysis. Finally, our study only has two time points: MRI data at baseline and the 12-month follow-up. In other words, we did not collect data immediately following the completion of the intervention. These limitations may affect the external validity of the study, and deserve to be examined further.

## Conclusion

In summary, our findings may contribute to an understanding of the mechanism of normal aging in the human brain and help to distinguish the different effects of multi-domain and single-domain cognitive training interventions in healthy older adults. Specifically, multi-domain cognitive training demonstrated more advantages in visuospatial/constructional, attention, and delayed memory abilities, while single-domain cognitive training primarily benefited immediate memory ability. In the end, multi-domain cognitive training may represent the best opportunity to improve the cognitive function of healthy older adults.

## Author Contributions

Conceived the study: CL, JW. Designed the experiment: CL, JW, LJ, XC, TL. Performed the experiment: LJ, XC, TL, WL. Analyzed the data: LJ, YT, WL, RCC. Wrote the manuscript: LJ, XC, TL, RCC, CL, JW. All authors provided critical revisions to the manuscript and approved the final version of the article.

## Conflict of Interest Statement

The authors declare that the research was conducted in the absence of any commercial or financial relationships that could be construed as a potential conflict of interest. The reviewer KRB and handling Editor declared their shared affiliation, and the handling Editor states that the process nevertheless met the standards of a fair and objective review.

## Footnotes

^1^http://surfer.nmr.mgh.harvard.edu
